# Assessing the Complications of Monopolar Transurethral Resection of the Prostate (M-TURP) Using Clavien-Dindo Complications Grading System

**DOI:** 10.4314/ejhs.v32i3.17

**Published:** 2022-05

**Authors:** Lijalem Mekonnen Geremew, Samuel Amare Gelaw, Andualem Deneke Beyene

**Affiliations:** 1 Urology Unit, School of Medicine, College of Health Sciences, Addis Ababa University

**Keywords:** Complications, Clavien-Dindo, Monopolar, Transurethral Resection of the Prostate (TURP)

## Abstract

**Background:**

Benign prostatic hyperplasia (BPH) is one of the most common diseases of ageing men, which increase starting from age 40. Monopolar transurethral resection of the prostate (M-TURP) is the gold standard surgical treatment for BPH between 30 to 80cc. This study is designed to assess complications of TURP based on the Clavien-Dindo post-op complication grading system.

**Methods:**

A descriptive prospective study of prevalence of complications of M-TURP from May1, 2019 to April 30, 2021 in Tikur Anbessa Specialized Hospital. In this study, 174 patients were assessed pre-operatively, intra-operatively and post-operatively. All BPH patients with bothersome LUTS, bladder stone, recurrent UTI, recurrent acute urinary retention (AUR), renal insufficiency, or failed medical therapy had undergone TURP. We collected it by revising patient's icare/charts and cell phone call.

**Results:**

About 174 patients were studied with mean age were 63 years. Intra op complications were noticed in seventeen (9.8%) patients, the most common one was prostate capsule perforation seen in 9 (5.2%) cases followed by severe bleeding in 3(1.7%) patients which needs transfusion and urethral injury. Urinary tract infections developed in eleven (7.8%) of patients. Bulbar urethral stricture and bladder neck contracture developed in 9 (5.2%) and 3 (1.7%) patients each, respectively. Re TURP was done for 7 (4%) of patients.

**Conclusion:**

In this study, the overall complication rate of TURP with Clavien-Dindo grading system was 29.3%. Around 96% of the complications were Clavien-Dindo grade I, II and III; managed conservatively or with minimally invasive surgery. This shows M-TURP is a relatively safe procedure.

## Introduction

Benign prostatic hyperplasia (BPH) is a common condition in men after the age of 40 years. The incidence rises with age. It is a significant cause of lower urinary tract symptoms (LUTS) causing bladder outlet obstruction (BOO) (1). Its prevalence and severity increase starting from the age of 40 years, reaches 50% after age 60years and 90% after 85 years (2). About 50% of all men who have a histologic diagnosis BPH have moderate to severe lower urinary tract symptoms (LUTS) (3) and it is remarkably similar across cultures and ethnicity. The indications for treatment are bothersome LUTS, recurrent urinary retention, recurrent urinary tract infections (rUTIs), recurrent gross hematuria, obstructive uropathy and bladder stones (4). Conservative management, medical therapy, minimal invasive surgical therapies and major surgical therapies (open, TURP) are the management options (1).

Despite the development of new technologies with relatively fewer complications like LASER, monopolar TURP is still the gold standard surgical treatment for BPH between 30 to 80cc with an 80%–85% success rate (5). The irrigation fluids used for monopolar TURP include sterile distilled water, 1.5% or 3% glycine, 3.5% sorbitol, 5% mannitol, 1% urea and 5% dextrose water (6). Although monopolar TURP is one of the common endourology procedures being done routinely in TASH since 2006, there was only one study comparing its complications and outcome with suprapubic trans-vesical prostatectomy (STVP) in our hospital. To the best of our knowledge so far, there is no other study in our institution using the Clavien-Dindo tool showing the prevalence of its complications. This study is designed to assess the complications of TURP based on the Clavien-Dindo post-op complications grading system (box 1). Currently, it widely used for the reporting of complications related to urologic surgical interventions (7). It is a two-year prospective assessment of the magnitude and grades of complications of M-TURP intraoperatively and during the first 6 months postoperative follow-up period.

**Box 1:** Descriptions of Clavien-Dindo grading system

Grades	Definitions
I	Any deviation from the normal postoperative course without the need for pharmacologic treatment or surgical, endoscopic and radiologic interventions. Acceptable therapeutic regimens are drugs such as antiemetics, antipyretics, analgesics, diuretics, and electrolytes, and physiotherapy. This grade also includes wound infections opened at the bedside
II	Requiring pharmacologic treatment with drugs other than those allowed for grade I complications. Blood transfusions, antibiotics and total parenteral nutrition are also included
III	Requiring surgical, endoscopic, or radiologic intervention
IIIa	Intervention not under general anaesthesia
IIIb	Intervention under general anaesthesia
IV	Life-threatening complication (including central nervous system complications (excluding transient ischaemic attacks) requiring intermediate care/ICU
Iva	Single-organ dysfunction (including dialysis)
IVb	Multiorgan dysfunction
V	Death of a patient
Suffix “d”	If the patient suffers from a complication at the time of discharge, the suffix “d” (for disability) is added to the respective grade of complication. This label indicates the need for a follow-up to evaluate the complication fully

## Methods

The study was conducted from May1, 2019 to April 30, 2021 in Tikur Anbessa Specialized Hospital, College of health Sciences, Addis Ababa University. It is a descriptive prospective, hospital-based assessment of post M-TURP complications based on Clavien-Dindo complications grading system. The study population was all urology patients admitted from May1, 2019 to April 30, 2021.

From May 1, 2019 to April 30, 2021, 218 patients underwent TURP of which 44 patients were excluded (25 were missed from follow up, 7 were diagnosed for prostate cancer, 9 had concomitant bladder tumor, 3 had prior Urethral stricture). Therefore, 174 patients were included in the study. All patients were preoperatively investigated with complete blood count (CBC), urine analysis/urine culture, renal function test (RFT), serum electrolyte and abdomino-pelvic ultrasound.

All TURPs were done by urology residents under consultant urologists supervision using 26Fr resectoscope, monopolar electric current and continuous irrigation system using 5% Dextrose in Water (DW). All BPH patients with bothersome LUTS, bladder stone, recurrent UTI, recurrent acute urinary retention (AUR), renal insufficiency, or failed medical therapy had undergone TURP. All the necessary data was collected using a designed questionnaire that included the preoperative, intraoperative, and the first six months postoperative data. We collected it by revising patient's icare/ charts and cell phone call.

All patients took Ceftriaxone 1gm IV before spinal anesthesia and continued post op for 24 hours. All identified complications were classified based on the Clavien-Dindo post-op complication grading system (box 1). We used IBM SPSS version 26 to analyze the data and the results presented using percentages, tables, charts and graphs. Ethical clearance was granted by Addis Ababa University, College of Health Sciences, Dept. of Surgery Research and Publications committee and informed consent was taken from the patients.

## Results

**Figure 1:**
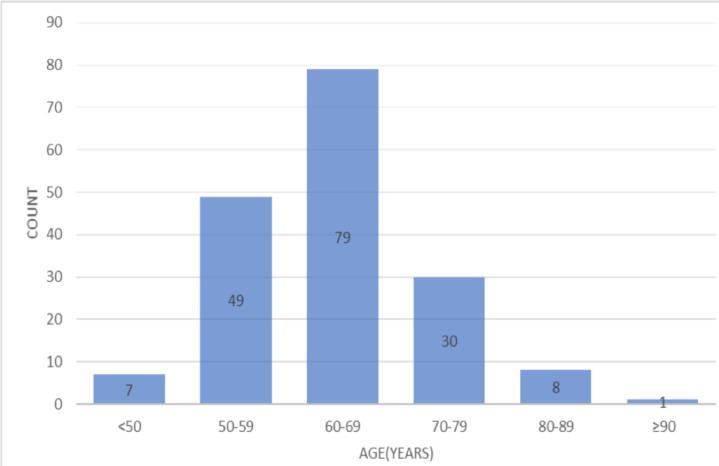
Age distribution of patients who underwent M-TURP (n=174)

**Figure 2:**
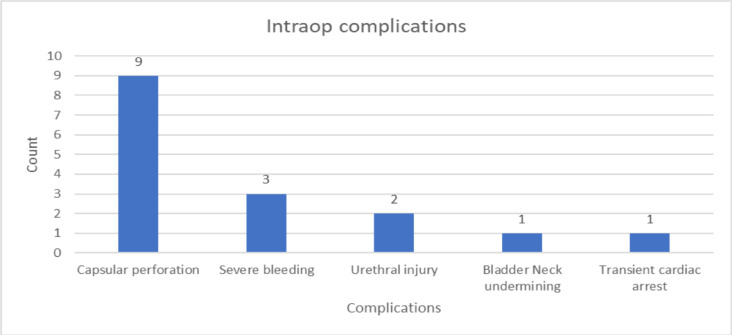
Comorbidities of patients who underwent M-TURP (n=174)

**Socio-demographic and pre-operative result**: The mean age was 63 years (range from 40 to 92years) (Fig. 1). Eighty-three (47.7%) patients had comorbidities: Hypertension (HTN) in 50(28.7%), diabetes mellitus (DM) in twenty-one (12.1%), neurologic diseases in fifteen (8.6%), retroviral infections (RVI) five (2.9%), cardiac diseases and asthma each in 3 (1.7%), chronic liver disease (CLD) in one (0.57%) patient (Fig. 2).

All patients presented with LUTS of 2–96months duration (mean of 21.6 months). Sixty patients (34.5%) had been catheterized preoperatively due to urinary retention or renal insufficiency for a mean duration of 10.8weeks (range of 2 - 208 weeks). The average prostate volume was 51.1cc (range 15–91cc) (Table 1). The most common indication (43.7%) for TURP was bothersome LUTS followed by recurrent AUR, failed medical therapy, renal insufficiency, bladder stone, recurrent UTI and hematuria (Table 2).

**Table 1:** Preoperative, intraoperative and postoperative conditions of M-TURP patients (n=174)

Description	Range	Mean	SD
Duration of LUTS (months)	2 – 96	21.6	18.513
Durations of preoperative transurethral catheterization (weeks) (n=60)	1 – 208	10.8	27.289
Prostate volume(cc)	15 – 98	51.1	17.683
Durations of surgery(minutes)	25 – 135	61.2	21.862
Durations of bladder irrigation(hours)	12 – 48	20.3	4.721
Durations of post op catheterization (hours)	16 – 240	48.8	33.367
Post-operative hospital stays (days)	1 – 13	2.1	1.395

**Table 2:** Indications for patient s who underwent M-TURP (n=174)

Indications	Frequency	Percent
Bothersome LUTS	76	43.7
Recurrent AUR	41	23.6
Failed medical treatment	31	17.8
Renal insufficiency	9	5.2
Bladder stone	4	2.3
Bleeding prostate	1	0.6
Bleeding BPH and renal insufficiency	1	0.6
Recurrent AUR and failed medical therapy	5	2.9
Recurrent AUR, recurrent UTI and renal insufficiency	6	3.4
**Total**	**174**	**100**

**Figure 3:**
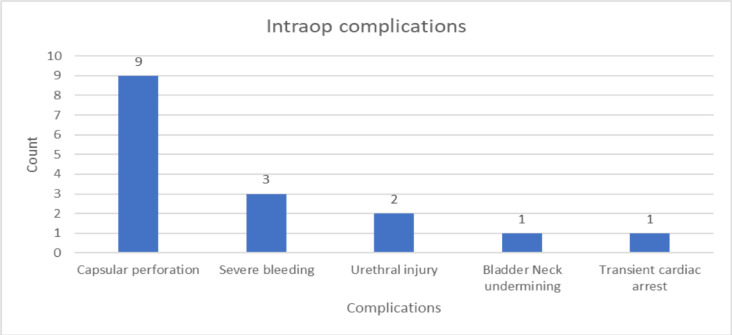
Intraoperative complications of patients who underwent M-TURP (n=174)

**Intra-op complications**: The mean duration of surgery was 61.2minutes with a range of 25–135minutes. Intra-operative complications were noticed in sixteen (9.2%) patients, the most common being prostate capsule perforation seen in nine (5.2%) cases. Three (1.7%) patients had significant bleeding. One patient was transfused intraoperatively with 1.5gm/dl post op hemoglobin drop. There was a decrement in hemoglobin of 2.5gm/dl and 3gm/dl in the other two patients; each transfused one unit of blood post operatively. Urethral injury occurred in two (1.1%) and one (0.6%) patient had bladder neck undermining. Transient cardiac arrest developed in one (0.6%) patient at the beginning of the procedure due to high spinal anesthesia (Fig. 3).

**Postoperative complications at first week, third month and sixth month**

**First week**: The average post-op bladder irrigation time was 20.3hours (range 12 to 48 hours) and duration of post-operative catheterization ranged from 16 to 240 hours (mean of 48.8hours). Most patients (94.3%) were discharged on their second post operative day (range 1^st^ to 13^th^ postoperative day). Seven (4.0%) patients failed trial of voiding after catheter removal. The stated causes were incomplete TURP in 4 (2.3%), clot retention, chips and detrusor muscle failure in one patient each. Three patients underwent second session of TURP and additional two sessions of TURP were done for one patient. One of the two remained on the catheter.

There was a transient hematuria and post op mild burning sensation in 153 (87.9%) patients and 152(87.4%) respectively. There was one sudden death (50 years old with no comorbidities) on his second post-op day while he was ambulating. During the first post-op week, around 147(84.5%) and 166 (95.4%) patients improved from their storage and voiding LUTS, respectively. One (0.6%) patient developed diffused necrotizing fasciitis for whom extensive debridement was done and he missed from follow up after he was referred to plastic and reconstructive clinic. There was also a new development of urge urinary incontinence eleven (6.9%) patients who had no this symptom preoperatively.

**Third month**: Additional eight patients improved from their transient worsening of post operative storage LUTS who had it during the first week post op period. Four (2.3%) patients were persistently suffering from urinary incontinence in the 3 months. Ten (5.7%) patients developed UTI; treated with PO antibiotics and improved.

**Sixth month**: During the 6 months postoperative follow up, 149(85.6%) patients improved from their LUTS. Eight patients developed worsened symptoms of frequency, nocturia, intermittency, stream and urgency. One patient improved from post-operative urge urinary incontinence. Three (1.7%) patients had persistent urge urinary incontinence. Nine (5.2%) patients developed urethral stricture for whom directly visualized incision of the urethra (DVIU) was done. Three (1.7%) patients developed bladder neck contracture (BNC). Three (1.7%) patients developed recurrence of voiding symptoms from residual adenoma and repeat TURP was done. One patient died due to a non-urologic cause.

**Overall complications**: The overall complications of MTURP were about 31.1% (Table 3). Two or more complications can occur in one patient.

**Table 3:** Over all complications of patients with M-TURP (n= 174) by Clavien-Dindo grading system

Grades	Complications	Number	% (n=174)
Grade I	Capsular Perforation	9	5.2
(n=19, 10.9%)	Failed trial of voiding (clot/chips/detrusor failure)	3	1.7
	Urge Urinary incontinence	3	1.7
	Bulbar Urethral Injury	2	1.1
	Bladder Neck undermining	1	0.6
	Transient cardiac arrest	1	0.6
Grade II	UTI	11	6.3
(n=14, 25.8%)	Bleeding requiring transfusion	3	1.7
Grade III	Post-op urethral structure	9	5.2
(n=19, 10.9%)	≥2 session of TURP	4	2.3
	BOO 2o Residual adenoma	3	1.7
	Bladder Neck contracture	3	1.7
Grade IV	Necrotizing fasciitis	1	0.6
(n=1, 0.6%)			
Grade V	Death	1	0.6
(n=1, 0.6%)			
	Total	54	31.1

## Discussion

In this study we have tried to assess the complications of MTURP using the Clavien-Dindo complications grading system. To the best of our knowledge such a study is not done in our set up and in Ethiopia as well. The complications of the procedure could be due to the age-related factors, preoperative duration, preoperative and post operative interventions, and comorbidities in patients. This study shows, most complications of MTURP are lower Clavien-Dindo grades (Grades I- III) which can be managed either conservatively or with minimally invasive surgery.

The younger age population and lower comorbidities like other low- and middle-income countries (Nigeria, Turkey and India) (6, 8, 9, 10) may have contributed to the lower grades of complications

The average prostate volume which affects outcome is different in several studies made in different countries (6, 8, 9, 11). The indications for TURP differ according to duration of the problem and prior interventions if any have impact on the outcome of the procedure. Patients presenting with renal insufficiency and prolonged catheterization will have higher likelihood of complications. Patient presentation in our study is similar to the reviewed studies (4, 6)

A prolonged duration of MTURP can lead to some complications. The duration of the procedure in this study is comparable to the study done in South Korea, Turkey and India (9, 11, 12).

Capsular perforation is the commonest intra operative complications and is higher than studies from Germany and India which could be due to difference in the set up but is comparable to a study in done another African set up (4, 9). Severe bleeding with transfusion is also higher in our study (4,9).

The post-operative duration of catheterization which could lead to complications later is longer in our study (13, 14). Some patients developed urinary retention after catheter removal during the first post-operative period due to incomplete TURP, clot, chips and detrusor muscle effect.

Transient hematuria post operatively and mild burning sensation are the commonest complications which is similar to other studies. Mortality in our study is low but is a little higher than mortalities in other studies in Germany and India (4, 9). The higher rate of sequential improvement of LUTS during the study period is encouraging and is comparable with other studies (2).

Post-operative acute urinary retention improves in most patients but one patient with 4 years of preoperative catheterization remained on catheter until the end of the follow up. This could be due to bladder decompensation due to the prolonged catheterization (11). Some patients may need repeated sessions of TURP and in this study we could not tell whether this is due to technical issue or prostate size. Unexpected complications like diffuse necrotizing fasciitis which requires extensive debridement may develop. This could be due to minor trauma of the procedure in the perineum. Some patients develop persistent urge urinary incontinence which shows the importance preoperative counseling. In this study, UTI has the highest complication rate which is expected due to the higher number of patients with prolonged catheterization.

Complications arising due to trauma of the procedure such as Urethral stricture and Bladder neck undermining, surprisingly have no difference (9). Bulbar urethral stricture and bladder neck contracture can occur due to the trauma of MTURP or pre and post operative catheterization. These complications can be treated with direct vision incision of urethra (DVIU) and bladder neck incision (BNI) with local and spinal anesthesia respectively (9, 11, 16). Though a quarter of patients develop some kind of complication of MTURP, the majority are lower grade complications according to the Clavien-Dindo complications grading system. This shows M-TURP is a relatively safe procedure for our set up.
